# Factors Influencing Practitioner Choice in Nonparticipation in Medical Assistance in Dying

**DOI:** 10.1093/geroni/igaa057.133

**Published:** 2020-12-16

**Authors:** Janine Brown, Donna Goodridge, Lilian Thorpe

**Affiliations:** 1 University of Regina, Saskatoon, Saskatchewan, Canada; 2 University of Saskatchewan, Saskatoon, Saskatchewan, Canada

## Abstract

Canadians aged 65 and older compromised 79% of the individuals who chose a medically assisted death between January 1 – October 31, 2018. Despite public approval for MAID, and positive professional reception, few practitioners are participating in MAID care and the underlying factors influencing this require exploration. What are the factors considered by practitioners when contemplating MAID participation? Interpretive Description guided a qualitative, exploratory project. Data included semi-structured interviews with 35 practitioners who currently did not participate in MAID, interviewer field notes and reflective content. Data was analyzed through open coding, a constant comparative approach and thematic analysis. Participants are contemplating numerous endogenous and exogenous factors in determining care participation. The endogenous factors included philosophy of care, MAID/faith/spirituality congruence, the conceptualization of duty, comfort with death and previous life experiences. Within these factors, participants described knowing if MAID participation was, or was not, possible through a process of self-reconciliation. Those who thought MAID care participation may be possible, but yet were not participating, intentionally contemplated numerous exogenous factors including risk, time and practice factors, patient, family and community considerations, system structures, and visibility. Non-participation in MAID care is important to understand to support the self-determination of older adults who may consider MAID. Contemplating involvement in MAID care is complex with both numerous factors influencing practitioner choice. Practitioners require care options, safe passage, respect, model of care clarity, removal of practice barriers, open conversations, enhanced education opportunities and time to facilitate safe, supportive work environments and client care.

